# Homozygosity Mapping on Homozygosity Haplotype Analysis to Detect Recessive Disease-Causing Genes from a Small Number of Unrelated, Outbred Patients

**DOI:** 10.1371/journal.pone.0025059

**Published:** 2011-09-20

**Authors:** Koichi Hagiwara, Hiroyuki Morino, Jun Shiihara, Tomoaki Tanaka, Hitoshi Miyazawa, Tomoko Suzuki, Masakazu Kohda, Yasushi Okazaki, Kuniaki Seyama, Hideshi Kawakami

**Affiliations:** 1 Department of Respiratory Medicine, Saitama Medical University, Moroyama, Saitama, Japan; 2 Department of Epidemiology, Research Institute for Radiation Biology and Medicine, Hiroshima University, Hiroshima, Hiroshima, Japan; 3 Division of Functional Genomics and Systems Medicine, Research Center for Genomic Medicine, Research Center for Genomic Medicine, Saitama Medical University, Hidaka, Saitama, Japan; 4 Division of Translational Research, Research Center for Genomic Medicine, Research Center for Genomic Medicine, Saitama Medical University, Hidaka, Saitama, Japan; 5 Department of Respiratory Medicine, Juntendo University School of Medicine, Bunkyo-ku, Tokyo, Japan; Tokyo Metropolitan Institute of Medical Science, Japan

## Abstract

Genes involved in disease that are not common are often difficult to identify; a method that pinpoints them from a small number of unrelated patients will be of great help. In order to establish such a method that detects recessive genes identical-by-descent, we modified homozygosity mapping (HM) so that it is constructed on the basis of homozygosity haplotype (HM on HH) analysis. An analysis using 6 unrelated patients with Siiyama-type α1-antitrypsin deficiency, a disease caused by a founder gene, the correct gene locus was pinpointed from data of any 2 patients (length: 1.2–21.8 centimorgans, median: 1.6 centimorgans). For a test population in which these 6 patients and 54 healthy subjects were scrambled, the approach accurately identified these 6 patients and pinpointed the locus to a 1.4-centimorgan fragment. Analyses using synthetic data revealed that the analysis works well for IBD fragment derived from a most recent common ancestor (MRCA) who existed less than 60 generations ago. The analysis is unsuitable for the genes with a frequency in general population more than 0.1. Thus, HM on HH analysis is a powerful technique, applicable to a small number of patients not known to be related, and will accelerate the identification of disease-causing genes for recessive conditions.

## Introduction

Identification of susceptible genetic loci is of great importance for understanding the underlying mechanisms of a number of diseases, and thus aiding the development of their treatment. Whole-genome association studies using individuals not known to be related have been very successful for the analysis of common diseases [1], while linkage-based approaches have identified a number of genes with large effect sizes [2]. More lately, greater attention has been directed to diseases that cannot be investigated using these approaches, either because of the difficulty in collecting a large number of samples, or in finding a sizeable family with the disease [3]. Such diseases include those caused by multiple rare genetic variants or by genes with low penetrance or with effects that become apparent only in the elderly [4]. For unraveling the causes of such diseases, there is the need for an approach that is effective in the context of a small number of patients not known to be related.

The homozygosity mapping (HM) method was developed to identify a disease-causing gene through analyses of patients from inbred families [5]. This principle was later expanded and applied to patients from outbred families [6], [7]. Moreover, the use of SNP data from genome-wide analyses has increased the sensitivity of the detection [8], [9]. However, because the algorithm employed in HM is highly vulnerable to genotyping errors, an appropriate correction for such errors is required [10].

In contrast, the homozygosity haplotype (HH) method [9] is an imputation-free method for determining haplotypes, because it uses only a fraction of SNP genotyping data. When a region of conserved homozygosity haplotype (RCHH) is observed in different individuals, there is a reasonable possibility that these individuals share an identical-by-descent (IBD) fragment in 1 or both strands of the homologous chromosomes. The algorithm is robust to genotyping errors and thus requires very little or no correction for genotyping errors.

During a previous study that aimed to identify a disease-causing gene for amyotrophic lateral sclerosis (MIM 613435) [11], we encountered 2 unrelated patients who shared the same homozygous mutation in the *OPTN* gene (MIM 602432). In addition, the region of DNA encompassing the gene contained a number of SNPs that were homozygous in both patients (a runs of homozygous SNPs [RHS] [10]). Further, the RHS was contained in a 0.9-Mb region of conserved HH (RCHH) [9]. In contrast, the length of RCHH shared between either of the 2 patients and each of the 85 control subjects was shorter than 0.9 Mb. We therefore concluded that these 2 patients are very likely shared the disease-causing IBD gene [11]. We considered that the reasoning had a general application and the presence of a long RCHH that contains an RHS strongly suggested the presence of an IBD fragment. We then encoded this reasoning into a computer program, thereby establishing HM on HH analysis. Here, we show here that this is a powerful method that can identify susceptible loci by identifying homozygous IBD fragments from a small number of outbred patients.

## Methods

### Ethics Statement

This study was approved by the Institutional Review Boards of Saitama Medical University, Tokyo University, and Juntendo University. All patients involved in the current study provided written informed consent.

### HM on HH analysis

HM on HH analysis is a combination of HM analysis [5], [10] employing controls and HH analysis [9] employing controls. The analysis does not presume that the patients are from inbred families, and can be performed on patients from the general population. It searches for an RHS overlap that is contained in an RCHH (see below). A candidate region thus obtained may contain a recessive disease-causing gene.

### Most recent common ancestor (MRCA)

**Figure 1:**
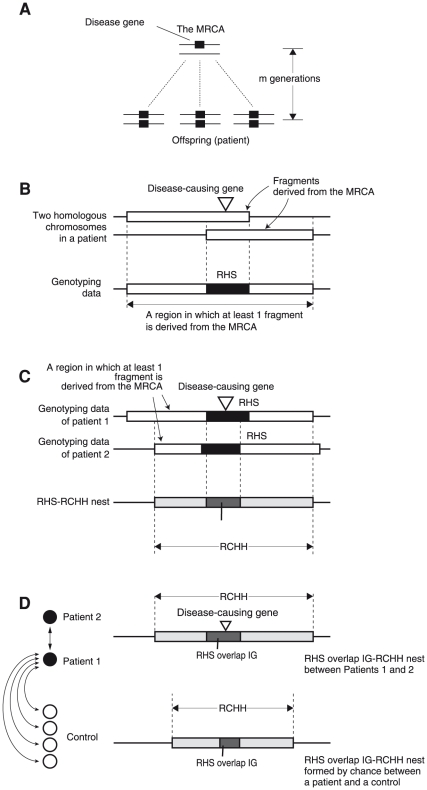
Structures formed by the fragments derived from the MRCA. (A) The MRCA has a single copy of the disease-causing gene. The gene is segregated to the patient through both the maternal and paternal lines, and thus the patients are homozygous for the disease-causing gene. (B) Each of the homologous chromosomes in the patient has a fragment derived from the MRCA. All SNPs in the overlap are homozygous, forming an RHS [10]. The union of the fragments generates “a region in which at least 1 fragment is derived from the MRCA.” (C) Assume that there are 2 patients. The genotypes of these patients are identical in the RHS overlap, forming an RHS overlap IG. The overlap of “regions in which at least 1 fragment is derived from the MRCA” forms an RCHH [9]. This RCHH therefore contains the RHS overlap IG. This nested structure is hereby called an RHS overlap IG-RCHH nest. (D) The 2 patients are compared with subjects from a general population (controls). An RHS overlap IG-RCHH nest may be formed between patients 1 or 2 and each of the controls due to a coincidence in the SNP genotype. However, the RHS overlap IG-RCHH nest between the patients is likely to be longer than any of the RHS overlap IG-RCHH nests accidentally formed between a patient and a control.

For patients sharing a disease-causing gene, the most recent common ancestor (MRCA) is the most recent ancestor from whom they inherited the recessive disease-causing gene (**Figure 1A**). Therefore, in the patients, the disease-causing gene is IBD. HM on HH analysis identifies 2 or more patients who are homozygous for this gene.

### Structure formed by the IBD fragments

The IBD fragments generate characteristic regions in the genotyping data both in a single patient and between 2 patients.

In a single patient, the overlap of 2 IBD fragments forms an RHS if its length is greater than the RHS cutoff (**Figure 1B**) [10]. Between 2 patients, RHSs can form an overlap (RHS overlap, hereafter). In the RHS overlap, the genotypes of both subjects are identical, forming an RHS overlap in which 2 subjects share an identical genotype (RHS overlap IG, hereafter) (**Figure 1C**). In addition, the overlap of the “region in which at least 1 fragment is derived from the MRCA” generates an RCHH if its length is greater than the RCHH cutoff (**Figure 1C**) [9]. The RHS overlap IG is contained in the RCHH, and the structure is hereby called the RHS overlap IG-RCHH nest. An RHS overlap IG-RCHH nest may be formed by chance between a patient and a control due to a coincidence in the SNP genotype. However, the RHS overlap IG-RCHH nest between the patients is likely to be longer, both in the size of the RHS overlap IG and in the size of the RCHH, than that formed by chance between a patient and a control (**Figure 1D**). Consequently, if we detect an RHS overlap IG-RCHH nest between 2 patients and it is longer than any of that detected between each patient and each control both in the size of the RHS overlap IG and in the size of the RCHH, the RHS overlap IG-RCHH nest is likely to suggest the presence of the IBD fragments in these 2 patients.

### HM on HH analysis

**Figure 2:**
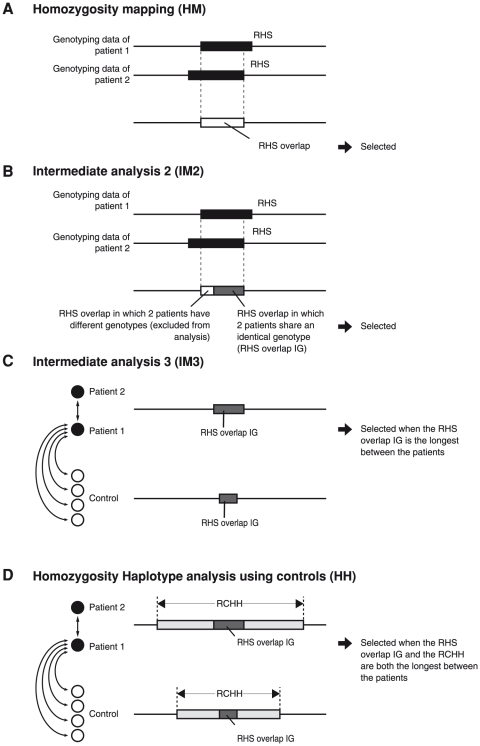
Steps of HM on HH analysis. HM on HH analysis is composed of 4 steps that are serially performed. (A) Homozygosity mapping (HM). The RHSs are determined for each patient, and the RHS overlaps are obtained. (B) Intermediate analysis 2 (IM2). The RHS overlap IGs are determined. (C) Intermediate analysis 3 (IM3). The RHS overlap IGs are compared. The RHS overlap IG is selected as a candidate region when the RHS overlap IG is the longest between the patients. (D) HH analysis using controls. RHS overlap IG-RCHH nests are compared. The RHS overlap IG is selected as a candidate region when the RHS overlap IG and the RCHH are both the longest between the patients.

HM on HH analysis searches for the RHS overlap IG-RCHH nest. The analysis is composed of 4 steps. Step 1: HM. The RHSs are obtained, and the RHS overlaps are selected as candidate regions for a disease-causing gene (**Figure 2A**) [10]. Step 2: Intermediate analysis 2 (IM2). RHS overlap IGs are selected as candidate regions (**Figure 2B**). Step 3: Intermediate analysis 3 (IM3). For each SNP position contained in an RHS overlap IG detected in Step 2, the presence of an RHS overlap IG between a patient and a control is investigated. When the RHS overlap IG between the 2 patients is longer in size than any of those between a patient and a control, it is selected as a candidate region (**Figure 2C**). Step 4: HH analysis using controls. The RHS overlap IG-RCHH nest is determined between 2 patients. For each SNP position contained in the RHS overlap IG in the RHS overlap IG-RCHH nest, the presence of an RHS overlap IG-RCHH nest formed between a patient and a control is investigated. When the RHS overlap IG between the 2 patients is longer in length than any of those formed between a patient and a control, and the RCHH between the 2 patients is longer in length than any of those formed between a patient and a control, the RHS overlap IG is selected as a candidate region (**Figure 2D**).

### Parameter values

The parameter values used in the current study were as follows. The RHS cutoff was 1.2 centimorgans. At this cutoff, the total length of the regions falsely identified as RHSs was less than 1.5 centimorgans in a genome-wide search [10]. Meanwhile, 8.4% of the total length of RHSs fail to be identified as RHSs when the MRCA occurred 20 generations ago; 25%, 40 generations ago; 42%, 60 generations ago; 57%, 80 generations ago, and 69%, 100 generations ago (**Figure S1A**). Before detecting the RHSs, a genotyping error correction algorithm was applied, with the suspected genotyping error rate set at 0.006 [10]. The RCHH cutoff was 0.0 centimorgans; thus, a match of HH of any length was considered to be an RCHH.

### Human subjects

#### Patients with Siiyama-type α1-antitrypsin deficiency (MIM 107400.0039)

Siiyama-type α1-antitrypsin deficiency is a rare recessive disease in Japan [12]. Whole-genome high-density SNP array genotyping data of 6 patients [10], who were not related and lived in different areas of Japan, were used in the current study. All patients provided written informed consent. The maximal likelihood estimates of the generational distance of the MRCA for each pair of patients ranged between 5 and 74 (median 61) generations.

#### Control subjects

The whole-genome high-density SNP genotyping data of 198 healthy Japanese subjects from the general population were provided by Prof. Tokunaga, Tokyo University. Additionally, the SNP genotyping data of 116 JPT (Japanese in Tokyo) subjects was obtained from the HapMap3 release 28 (http://hapmap.ncbi.nlm.nih.gov/), and data corresponding to the SNPs employed in the Genome-Wide Human SNP Array 6.0 were extracted. From these 314 subjects, we chose 261 subjects based on the number of SNPs genotyped (the number of successfully genotyped SNPs for the selected 261 subjects ranged between 707041 and 903804). These 261 subjects were randomly assigned as controls (200 subjects), as participants in a test population (20, 40, or 60 subjects), and a subject who served as the MRCA. The number of controls used was determined because 200 was the largest round number of controls that could be used. The number of the patients in the test population was determined so that the largest test population had 10 times the number of patients with Siiyama-type α1-antitrypsin deficiency; it was believed that this number was suited for demonstrating the power of the analysis and for enabling an easy interpretation of the analysis results.

### Genotyping

SNP genotyping was performed using the Genome-Wide Human SNP Array 6.0 (Affymetrix).

### Synthetic data

The synthetic genotyping data of a patient who shared 2 IBD fragments that contain a disease-causing gene were made as follows: (i) A subject was randomly chosen from the 261 subjects (see above) to serve as the MRCA. (ii) An SNP was randomly chosen from an autosomal region and was considered to mark the position of the disease-causing gene. (iii) The range of the chromosomal region that contained the SNP and was inherited by the patient from the MRCA was calculated according to the Haldane's Poisson process model [13]. (iv) Step (iii) was repeated for the second fragment. (v) The genotyping data of the patient corresponding to the regions that were obtained at steps (iii) and (iv) were replaced with those of the MRCA.

### Variables investigated in HM on HH analysis of a population

The variables investigated were the number of subjects in the test population (20, 40, and 60), proportion of patients in the test population (0, 5, 10, 15, 20, 25, and 30%), generational distance of the MRCA (20, 40, 60, 80, and 100 generations), and the gene frequency in the general population (0.0, 0.05, and 0.1). A gene frequency of 0.0 was considered to represent a rare variant, while gene frequencies of 0.05 and 0.1 were considered to represent common variants.

### Computer program

The program was written in the Ruby programming language (http://www.ruby-lang.org/en/) with an extension library written in the C programming language (http://gcc.gnu.org/). The program was executed on a MacPro computer that ran on MacOS X 10.6.

### Program

HM on HH program is available at Homozygosity Haplotype Analysis Web site, http://www.hhanalysis.com

## Results

### HM on HH analysis in patients with Siiyama-type α1-antitrypsin deficiency

**Figure 3:**
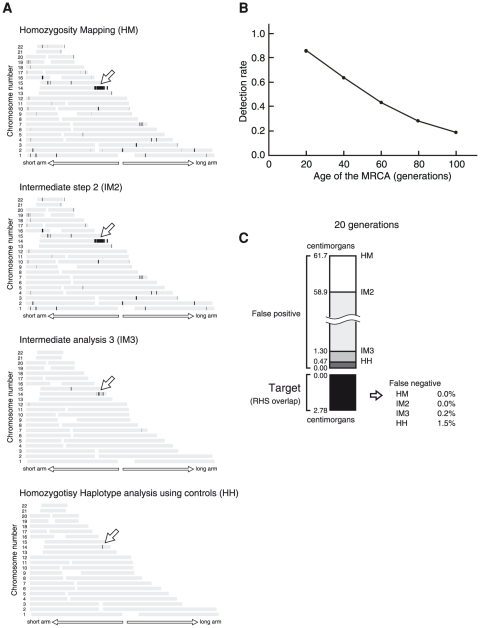
HM on HH analysis of a pair. (A) Analysis of patients 1 and 2 with Siiyama-type α1-antitrypsin deficiency. The position of the disease-causing gene (*SERPINA1*) is indicated by an arrow. HM on HH analysis is composed of 4 steps that are sequentially performed. The regions selected after each step are shown as black bands. The total length of the regions selected at the end was 1.36 centimorgans. (B) The rate at which the RHS overlap was detected by the HM step (i.e., the first step of the analysis) was the major determinant of HM on HH analysis. The detection rate will be improved by genotyping more SNPs genomewide. (C) False positives and false negatives for each analysis. False negatives are decreased with the progression of the analyses. False negatives are very few: 1.5% of the RHS overlap detected by the HM analysis is falsely excluded by HM on HH analysis.

We tested the performance of HM on HH analysis by using the SNP genotypes of 6 unrelated patients with Siiyama-type α1-antitrypsin deficiency, a rare autosomal recessive disease in Japan caused by a founder mutation of the *SERPINA1* gene (MIM 107400) [12]. As controls, we employed the genotypes of 200 Japanese individuals from the general population. The results obtained after each of the 4 steps that compose HM on HH analysis are shown for a pair of patients (**Figure 3A**). After the completion of the analysis, 2 closely located regions with a total length of 1.4 centimorgans were identified, 1 of which contained *SERPINA1* (**Figure 3A**). The results of the other 14 patient-pair combinations (note that 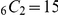) were similar: each combination identified candidate regions (total length: 1.2 to 21.8 centimorgans, median: 1.6 centimorgans) that contained *SERPINA1*. Using the genotyping data of only 2 patients, HM on HH analysis was able to narrow the position of the disease-causing gene to a very short chromosomal interval.

### HM on HH analysis of a pair of synthetic patients

We further examined the performance of HM on HH analysis of a pair of patients using synthetic data. We investigated the MRCA at 5 different generational distances (20, 40, 60, 80 and 100 generations). For each distance, we employed 60 randomly selected subjects, so that a total of 1770 pairs (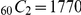) were constructed. Each pair was investigated for 100 randomly selected SNP locations, which were assumed to be the location of a disease-causing gene. The number of trials was thus 177000 (1770 combinations×100 SNPs) for each generational distance.

Detection of the region containing the RHS overlap depended on the generational distance of the MRCA (**Figure 3B**). This was a major limitation of HM on HH analysis: at the HM step, only RHSs that were longer in length than the RHS cutoff were detected (**Figure S1A**) [10]. The detection will be improved by genotyping more SNPs at a genomewide level, which will allow the use of a smaller RHS cutoff value (**Figure S1B**). Once an RHS overlap was detected at the HM step, HM on HH analysis rarely failed to track it (**Figure 3C**): for the MRCA that occurred 20 generations earlier, the RHS overlap was falsely excluded (false negative) in only 1.5% of the cases, while the falsely included areas (false positive) were reduced from 61.7 centimorgans after the HM step to 0.47 centimorgans after the completion of the HH step, indicating that a small false positive is a prominent feature of HM on HH analysis. Data for the other generations of the MRCA are presented in **Figure S2**.

### HM on HH analysis of a population

**Figure 4:**
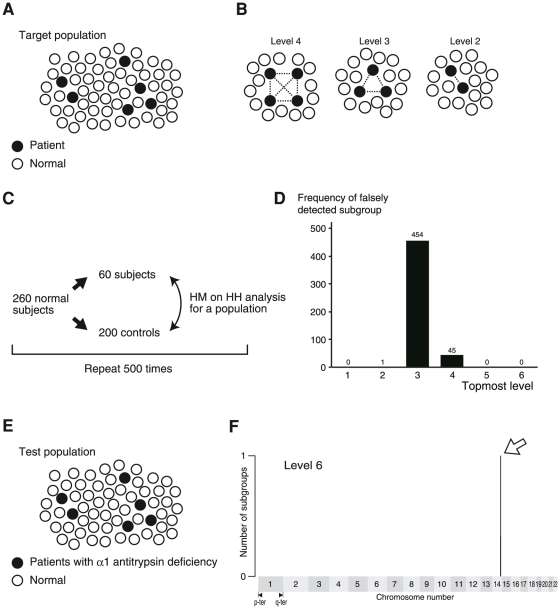
HM on HH analysis of a population. (A) Targets. Targets of HM on HH analysis of a population are populations containing multiple subjects sharing an IBD fragment. (B) Analysis level. At the level *n* analysis, a subgroup of *n* members, each pair of which shares an IBD fragment at the same position on the chromosome, are sought. (C) Scheme of the background determination. (D) Background. A subgroup(s) was falsely detected at level 3, 4, and 5 analyses. (E) A test population. The population is composed of 6 patients with Siiyama-type α1-antitrypsin deficiency (black circles) and 54 normal subjects (white circles). (F) Result. The horizontal position indicates the location on the autosomes, each of which is aligned from the p terminal (left side) to the q terminal (right side). A single subgroup was identified at the level 6 analysis, and the candidate region contained the *SERPINA1* gene. The members of the subgroup, which was the output on the computer console and thus is not shown here, were the 6 patients with Siiyama-type α1-antitrypsin deficiency.

HM on HH analysis of a population targets a population containing multiple patients sharing an IBD fragment (**Figure 4A**). This simulates a situation in which the population is a collection of patients with the same disease, and some of the patients share an IBD gene. We attempt to identify (1) a patient subgroup sharing an IBD fragment and (2) the chromosomal location of the shared IBD fragment. Here, we defined the analysis level: at analysis level *n*, the computer program searches for a subgroup consisting of *n* patients, any pair of which shares an IBD fragment at the same position on the chromosome (**Figure 4B**). To achieve the aims (1) and (2) as stated above, the program identifies (a) the topmost analysis level at which any subgroup is detected, (b) the members that are contained in the subgroup, and (c) the position of the IBD fragment on the chromosome.

First, we investigated the background signal that was detected in the general population (**Figure 4C**). For this purpose, we employed 260 normal subjects. Step (a): 260 normal subjects were randomly divided into a test population (60 subjects) and 200 controls. Step (b): HM on HH analysis of a population was performed. Steps (a) and (b) were repeated 500 times. The histogram of the topmost analysis level, at which any subgroup was detected (**Figure 4D**), demonstrated that a subgroup could be falsely detected (i.e., false positive) in the level 4 analysis and in an earlier analysis level. Conversely, when a positive result was obtained in the level 5 analysis or in a later analysis, a subgroup sharing an IBD fragment was likely to be detected. Next, we investigated a test population comprising 6 unrelated patients with Siiyama-type α1-antitrypsin deficiency and 54 normal subjects (**Figure 4E**). A subgroup was detected at level 6 (**Figure 4F**); the members of the subgroup were the 6 patients with Siiyama-type α1-antitrypsin deficiency. The candidate region, 1.2 centimorgans in width, was located on chromosome 14 and contained the *SERPINA1* gene. HM on HH accurately isolated a subpopulation that accounted for only 10% of the population and identified the position of an IBD fragment on the chromosome.

### HM on HH analysis of a population containing synthetic patients

**Figure 5:**
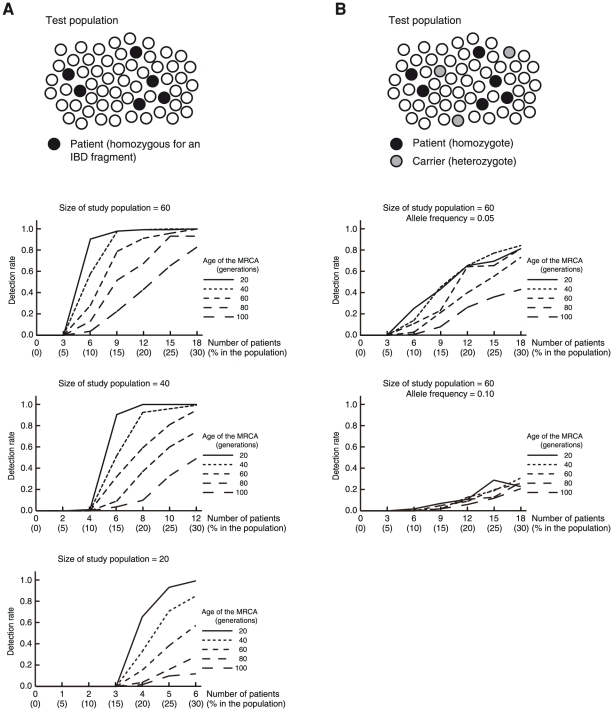
HM on HH analysis of a population performed on populations containing synthetic patients. (A) Scheme of the analysis and Result. The test population is composed of patients homozygous for a gene identical by descent (black circles) and subjects who do not have the gene (white circles). The horizontal line of each graph indicates the number (percentage) of patients homozygous for the gene in the test population. (B) Scheme of the analysis and Result for a gene that is widely shared in the general population. The test population is composed of patients homozygous for a gene identical by descent (black circles), subjects heterozygous for the gene (gray circles), and subjects who do not have the gene (white circles). In the case that the gene was widely shared in the general population (the frequency of the allele of the disease-causing gene was 0.05 and 0.1), the detection rate was decreased.

To study the performance of HM on HH analysis in more detail, we studied test populations containing synthetic patients. The synthetic patients (5, 10, 15, 20, 25, and 30% of the members of the population) were homozygous for the IBD fragment derived from MRCAs at generational distances of 20, 40, 60, 80, and 100 generations. For each combination of these parameters, the analysis was repeated 100 times by changing the disease-gene location, which was randomly selected from the SNP positions on the autosomes. The analysis was considered successful when (1) only a single candidate region was detected in the topmost level that detected any subgroup, and (2) the candidate region contained the locus of a disease-causing gene. The rates of successful trials (detection rate) were graphed for populations with 60, 40, and 20 subjects (**Figure 5A**). The results demonstrated that HM on HH could identify a subpopulation sharing an IBD fragment that accounted for only a small fraction of the population.

The analysis described above assumed that the frequency of the allele containing the disease-causing gene was 0.0 in the general population. However, the disease-causing gene may be a common variant. We investigated the performance of HM on HH analysis when the frequency of the disease-causing gene in the general population was 0.05 or 0.1 (**Figure 5B**). The results indicated that the performance was severely degraded for a frequency of 0.1. HM on HH analysis was considered to work well for a frequency <0.1. Therefore, the HM on HH analysis targets a recessive gene that is the cause of a disease, in which less than 1% of the people in the general population are homozygous for the gene and thus may suffer from the disease somewhere in their lifetime.

### Analysis without utilizing HH information

Analyses similar to HM on HH analysis may be performed by stopping the analysis after the HM, IM2, or IM3 steps (**Figure 3A**). When stopping after either the HM or the IM2, the program ran out of memory from the explosion in the numbers of subgroups that resulted from a large amount of false positives. When stopping after the IM3 step, the detection rate was much lower than that after the HH analysis, because of a greater amount of false positives (**Figure s3**). These results demonstrated that the small amount of false positives attained by the HH step is important for the performance of HM on HH analysis of a population.

## Discussion

In the current report, we demonstrated that HM on HH is able to narrow the candidate region for a disease-causing gene to a very small chromosomal interval either by employing 2 outbred patients sharing an IBD fragment, or by using a small population in which 10%≤ of the patients share an IBD fragment. Haplotype information obtained from the region that flanks the RHSs was the component of the HM on HH analysis that enabled them. By using the HM on HH analysis, genes with a recessive trait are exploited in the very early stage of a project attempting to identify a disease-causing gene.

It has been reported that HM is able to identify a candidate region from as few as 3 inbred patients [5], [8]. Although this number is small, the clinical characteristics often do not provide information sufficient for selecting 3 patients who may have an IBD gene. Furthermore, the total length of the candidate regions detected by HM is usually large [8], [11], which necessitate an enormous effort for an in-depth search of the regions. HM on HH analysis offers the advantage of being capable of using only 2 patients to obtain a relatively narrow candidate region, typically it is a few centimorgans in length. This may enable novel strategies for identifying disease-causing genes. One such strategy is to collect several patients who are likely to share a fragment IBD, identify the candidate regions by a pairwise comparison, and scrutinize all of these regions by high-throughput sequencing [14].

A small number of founder mutations often largely accounts for the occurrence of a recessive disease or its predisposition. Examples are α1-antitrypsin deficiency and cystic fibrosis in Europeans [15], [16], and Gaucher disease and Tay-Sachs disease in Ashkenazi Jews [17]. The cause of the prevalence may be heterozygote advantage, a founder effect, or genetic drift [17], [18]. Whatever is the cause, this suggests that the predominance of a limited number of founder mutations is worth taking into consideration in an attempt to search for disease-causing genes with a recessive trait. HM on HH analysis is suitable for pursuing the possibility.

The generational distance of the MRCA has a major effect on the performance of HM on HH analysis. In the analysis of pair of patients, the effect was large (**Figure 3B**). In the analysis of population, the effect was moderate (**Figure 5A**). Use of arrays with a greater number of SNPs will accomplish a better performance (**Figure S1B**). Data obtained using SNP Array 6.0 were investigated in the current study; they were considered suitable for the MRCA with a generational distance ≤60 generations (**Figures 3B** **and** **5A**). Founder populations that settled in recent centuries are amenable to the analysis. These include the French-Canadian population that settled in Quebec in the 17th century [19], [20], or the Icelandic population that was founded in the 10th century [21], because the generational distance of the MRCA may be less than 20–60 generations in many diseases.

Isolated populations may also be suitable for this analysis; in such population, a single IBD gene from an MRCA existed in a recent generation may predominate among patients with a specific disease. In many countries, there may be many geographical areas in which MRCAs for a disease-causing gene have a generational distance of 20–60 generations. A small number of patients that HM on HH analysis requires will make the analysis easily performable in small populations from such areas.

Inclusion of the subjects who share the IBD fragment degrades the performance of the analysis. The frequency of the gene in the control should be less than 0.1, i.e., less than 20% of the control subjects may be heterozygous for the gene and less than 1% of the control subjects may be homozygous for the gene. The analysis is not suitable for the common variants for the common diseases that are often the targets of the genome-wide association studies.

The calculation time of the HH on HH analysis is short. The analysis of a pair of patients is completed in a fraction of a second; the analysis for a test population of 60 subjects is completed in 15 seconds. Theoretically, an analysis of a study population of 60 subjects requires an investigation of 1.15×10^18^ subgroups. However, many of the comparison of 2 patients generate a result without any candidate region, and thus eliminate the need for investigating any subgroups containing a given pair. A small amount of false positive of HM on HH analysis enables an exhaustive search for the subgroups.

We used 200 controls in the current study, but it is possible to decrease this number with minimal loss in performance. When the analysis was performed with 100 controls, we found that the performance was only mildly decreased. Moreover, the International HapMap3 project (see International HapMap project Web page) has genotyped and released about 100 or more subjects for each of the 10 ethnic groups, and these data may be used for controls.

The RHS overlap IG-RCHH nest was selected when both the length of the RHS overlap IG and the length of the RCHH between the patients were both at the top. The criteria may be weakened to “top 1%,” “top 10%,” etc. However, we found that the condition of “at the top” worked best for almost all cases (data not shown). The current criterion is thus considered good for HM on HH analysis.

The RHS cutoff for the Genome-Wide Human SNP Array 6.0 was selected so that the total length of the false-positive RHS was acceptable (1.5 centimorgans per a patient). The equivalent RHS cutoff values for other high-density SNP arrays are 0.75 centimorgans for the Human Omni2.5 BeadChip (Illumina), 1.1 centimorgans for the Human1M-Duo BeadChip (Illumina), and 1.9 centimorgans for the GeneChip Human Mapping 500K Array Set (Affymetrix) [10].

In conclusion, HM on HH analysis used genetic information on both the RHS and the flanking regions, and thus detected the locus for a recessive, disease-causing genes with a very low background from a small number of patients. HM on HH analysis will accelerate the elucidation of the genetic causes of many diseases.

## Supporting Information

Figure S1 Errors in the HM. (A) The false positive rate is the ratio of the total length of RHSs that are falsely detected along the entire length of the autosomes. The false negative rate is the ratio of the total length of the autozygous segments (i.e., chromosomal regions in which both chromosomal fragments are IBD) that fail to be detected as RHSs along the total length of the autozygous segments. The false positive rate is dependent on the kind of high-density array and thus is shown for each array. 2.5 M, Human Omni2.5 BeadChip (Illumina); 1 M, Human1M-duo BeadChip (Illuina); SNP6.0: Genome-Wide Human SNP Array 6.0 (Affymetrix); 500K, 500K GeneChips Mapping Array Set (Affymetrix). m: the age of the MRCA. (B) Detection rates for each array. The figure corresponds to **Figure 3B**; this figure summarizes the theoretical calculation, while the result in **Figure 3B** is the result using the actual genotyping data.
(EPS)

Figure S2 Errors in HM on HH for a pair of patients. The data corresponding to those of **Figure 3C** for the MRCAs with a generational distance of 40, 60, 80, and 100 generations.
(EPS)

Figure S3 Result of the analysis stopped after the IM3 step. (A) Background. The background was observed to a higher analysis level than that for the HH analysis (compare with **Figure 4D**). (B) Detection rate. Positive results obtained at a level 7 analysis or higher were considered successful. (C) Detection rate. Positive results obtained at a level 9 analysis or higher were considered successful. Figures (B) and (C) correspond with those shown in **Figure 5A**. In both conditions, the detection rate was lower than those shown in **Figure 5A**.
(EPS)
